# Analysis of Softwood Lignans by Comprehensive Two-Dimensional Liquid Chromatography

**DOI:** 10.3390/molecules28248114

**Published:** 2023-12-15

**Authors:** Danil I. Falev, Ilya S. Voronov, Alexandra A. Onuchina, Anna V. Faleva, Nikolay V. Ul’yanovskii, Dmitry S. Kosyakov

**Affiliations:** Laboratory of Natural Compounds Chemistry and Bioanalytics, Core Facility Center “Arktika”, M.V. Lomonosov Northern (Arctic) Federal University, Northern Dvina Emb. 17, 163002 Arkhangelsk, Russia; i.voronov@narfu.ru (I.S.V.); a.onuchina@narfu.ru (A.A.O.); a.bezumova@narfu.ru (A.V.F.); d.kosyakov@narfu.ru (D.S.K.)

**Keywords:** comprehensive two-dimensional liquid chromatography, LC × LC, lignans, coniferous knotwood

## Abstract

Lignans constitute a large group of phenolic plant secondary metabolites possessing high bioactivity. Their accurate determination in plant extracts with a complex chemical composition is challenging and requires advanced separation techniques. In the present study, a new approach to the determination of lignans in coniferous knotwood extracts as the promising industrial-scale source of such compounds based on comprehensive two-dimensional liquid chromatography separation and UV spectrophotometric detection is proposed. First and second-dimension column screening showed that the best results can be obtained using a combination of non-polar and polar hydroxy group embedded octadecyl stationary phases with moderate (~40%) “orthogonality”. The optimization of LC × LC separation conditions allowed for the development of a new method for the quantification of the five lignans (secoisolariciresinol, matairesinol, pinoresinol, 7-hydroxymatairesinol, and nortrachelogenin) in knotwood extracts with limits of quantification in the range of 0.27–0.95 mg L^−1^ and a linear concentration range covering at least two orders of magnitude. Testing the developed method on coniferous (larch, fir, spruce, and pine) knotwood extracts demonstrated the high selectivity of the analysis and the advantages of LC × LC in the separation and accurate quantification of the compounds co-eluting in one-dimensional HPLC.

## 1. Introduction

Among the polyphenolic secondary metabolites of plants, a special place is occupied by lignans, which contain two phenylpropane units connected by the β-β alkyl-alkyl or other types of bonds (alkyl-alkyl and alkyl-aryl) in their structure. In the latter case, it is customary to use the term neolignans [1,2,3]. Lignans and neolignans are extremely widespread in nature and number more than a thousand currently known representatives, and this list is constantly expanding. Due to the unique biological activity of lignans possessing antioxidant, antitumor, hepatoprotective, and cardioprotective properties, the search for their natural sources and the development of analytical methods for the determination of these compounds in plant raw materials are becoming increasingly important.

Currently, compression wood (knots, roots) of coniferous trees, the active biosynthesis of lignans in which is a response to mechanical stress, is considered one of the most promising industrial sources of such compounds [4,5]. It is known that coniferous knotwood may contain up to 20% lignans, and their major representatives are 7-hydroxymatairesinol or HMR (spruce), nortrachelogenin (pine), and secoisolariciresinol (larch, fir). Matairesinol and pinoresinol are also present in large amounts [6]. In addition to the above-mentioned bioactive properties, these compounds are capable of being metabolized under the action of the gut microbiome with the formation of so-called enterolignans or mammalian lignans (enterodiol and enterolactone), which play an important biological role. In addition to these components, coniferous wood contains a number of other lignans, extractive substances of different classes (flavonoids, steroids, resin acids, etc.), and their glycosylated derivatives. This complicates the detailed analysis of the compression wood extracts and necessitates the use of advanced analytical techniques. 

Currently, the qualitative and quantitative analysis of lignans is carried out mainly by thin-layer (TLC), gas (GC), and high-performance liquid chromatography (HPLC), their combinations with various types of mass spectrometry (MS), and matrix-assisted laser desorption/ionization mass spectrometry (MALDI). TLC has mainly been used as an inexpensive adjuvant method in the study of various plant materials, including coniferous wood [7,8]. The high-performance version of this separation technique (HPTLC) was successfully used for the isolation, identification, and quantitative assessment of representatives of lignans, such as schisandrol A and schisandrol B, in various preparations of Chinese lemongrass (*Schisandra chinensis*) [9], phyllanthin, hypophyllanthin, niranthin, and nirtetralin in Phyllanthus species [10] as well as sesamin and sesamolin in sesame oil and its polyherbal formulations [11]. GC provides higher efficacy in lignan separation and is extensively used for identification and quantification purposes [12,13] in combination with MS detection. However, due to the low volatility and thermal lability of lignans, GC-MS requires preliminary derivatization of the analytes (typically, silylation) [14], which is a tedious time and labor consuming procedure that negatively affects the accuracy and reproducibility of the analysis. MALDI MS is distinguished by the simplicity of the sample preparation and tolerance to impurities; however, it cannot be easily combined with separation techniques [15]. 

In this regard, HPLC allowing the direct (without derivatization step) analysis of plant extracts can be considered a method of choice for the detection, identification, and quantification of lignans. The separation of the analytes is typically carried out on reversed stationary phases (e.g., octadecyl silica, C18) in gradient elution mode, providing sufficient resolution towards the major components of plant extracts. Thus, the authors of [16,17] reported the separation of isolariciresinol, secoisolariciresinol, anhydrosecoisolariciresinol, matairesinol, lariciresinol, hinokinin, arctigenin, and pinoresinol within 50 min and the possibility to resolve enantiomeric compounds using chiral stationary phases. In other noteworthy studies [18,19], the separation of ten Chinese lemongrass lignans was achieved within 40 min.

Various types of detection are used in combination with HPLC in lignan analysis, while UV spectrophotometry [7,16,17,18,19] and MS [6,20,21] are the most common techniques. The latter provides the highest selectivity and sensitivity and ensures the reliable identification of unknown compounds. At the same time, the quantification of analytes by HPLC-MS (especially with electrospray ionization that is extremely susceptible to matrix effects) requires corresponding analytical standards, which are hardly commercially available in the case of lignans. Apparently, HPLC-UV is less expensive and provides more possibilities for the standard-free semi-quantification of structurally close analytes containing the same chromophores due to their similar response factors and lower matrix effects in spectrophotometry when compared to MS. However, overcoming the low selectivity of UV detection requires achieving the highest chromatographic resolution between all the analytes and matrix components, which is difficult to achieve for such complex samples as plant extracts [7,17], even in ultra-performance (UPLC) analysis.

In our opinion, this issue can be resolved by implementing comprehensive two-dimensional liquid chromatography (LC × LC), which involves the consecutive separation of all the sample components on “orthogonal” (in chemical nature) first- (^1^D) and second-dimension (^2^D) columns [22]. Due to its high separation efficiency and peak capacity [23], LC × LC is increasingly used in the analysis of natural compounds, particularly plant secondary metabolites [22]. Examples include the determination of phenolic acids in wine [24] and flavonoids in plant extracts [25]. Specific lignans of Chinese lemongrass, schisandrins, were also successfully separated by LC × LC [26]. Despite this, there is still no information in the literature about the possibility of using LC × LC for the highly efficient analysis of softwood lignans.

The present study aimed to fill this gap and focused on developing an approach to the quantitative determination of lignans in coniferous knotwood extracts as the most important industrial-scale source of these compounds based on a combination of comprehensive two-dimensional liquid chromatography with diode array spectrophotometric detection.

## 2. Results and Discussion

### 2.1. Column Screening and Selection of LC × LC Conditions

A key factor in the LC × LC method development is the proper selection of a combination of stationary phases that ensures the most complete separation of the analytes. Considering the existing limitations on the composition (and thus elution power) of the sample solvent introduced into the ^2^D column, combinations of stationary phases with completely different retention mechanisms (for example, reversed and normal phase or hydrophilic interaction retention) were not used in our study. Instead, five reversed stationary phases, which have previously been proven to be effective in lignan separations [16,17,18,19], were chosen for further testing. They differ in the presence and nature of the embedded polar functional groups that affect the separation selectivity: (i) Shim-pack XR-ODS II—octadecyl-bonded silica, endcapped; (ii) Nucleodur C18 Isis—cross-linked octadecyl-bonded silica, endcapped; (iii) Nucleodur C18 Pyramid—octadecyl-bonded silica with hydrophilic (-CH_2_OH) endcapping; (iv) Nucleodur PolarTec—octadecyl-bonded silica with embedded polar (amide) groups, endcapped; and (v) Nucleodur PFP—pentafluorophenyl propyl-bonded silica, partially endcapped.

Considering the need to separate not only the most important lignans but also the matrix components affecting the target analytes’ UV detection, chromatographic runs were performed using the larch knotwood extract as a test sample. The six combinations of stationary phases (Table 1), including three pairs of ^1^D non-polar (Shim-pack XR-ODS II, Nucleodur Isis) and ^2^D polar functionalized (Nucleodur C18 Pyramid, Nucleodur PFP), and three pairs of ^1^D and ^2^D polar functionalized sorbents, were tested (Supplementary Figure S1).

The stationary phase orthogonality parameters (A_O_) and regression coefficient (R^2^) calculated from the measured retention times using “Asterisk” equations according to Camenzuli and Schoenmakers [27] are presented in Table 1. Since for the most efficient separation, the A_0_ and R^2^ values should be as high and as low as possible, respectively [28], the best results were observed for the following two combinations of stationary phases: ^1^D Shim-pack XR-ODS II—^2^D Nucleodur C18 Pyramid (Supplementary Figure S1a) and ^1^D Nucleodur PFP—^2^D Nucleodur C18 Pyramid (Supplementary Figure S1b). The attained orthogonality values for these pairs (39 and 40%, respectively) can be considered quite acceptable given that A_O_ > 43% already refers to high orthogonality [29]. Although both the A_O_ and R^2^ values for all the tested stationary phase combinations fell into rather narrow ranges (34–40% and 0.84–0.92, respectively) and, thus, good separation was achieved in all cases, the advantages of the two selected column pairs can be noticed, even visually, in Supplementary Figure S1. Considering the higher availability of the octadecyl stationary phases, the combination of the Shim-pack XR-ODS II (^1^D) and Nucleodur C18 Pyramid (^2^D) chromatographic columns was chosen for further studies.

**Figure 1:**
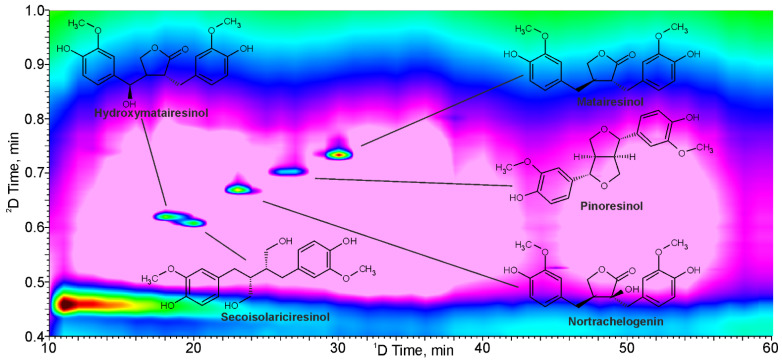
LC × LC-UV chromatogram (280 nm) of analytes model mixture (10 mg L^−1^) and chemical structures of the studied lignans.

Variation of the gradient elution profiles in both the ^1^D and ^2^D dimensions allowed the establishment of the elution programs with water (A) and acetonitrile (B), both acidified with 0.1% of formic acid, as mobile phase components: ^1^D—start from 15% B with linear ramp to 65% B during 60 min; ^2^D—start from 20% B with linear ramp to 90% B during 0.75 min, 20% B from 0.75 to 1.00 min for column equilibration. They ensured the achievement of the maximum distribution of the detected compounds in the 2D chromatogram (Supplementary Figure S1a), their fast elution (within 1 min) from the ^2^D column, and the full separation of lignans with similar retention on the reversed-phase columns—HMR and secoisolariciresinol (Figure 1). Since the maximum absorption of all the tested lignans was observed at 280 nm (Figure S2), the construction of two-dimensional chromatographic plots and the quantification were carried out at that wavelength.

### 2.2. Quantitative Analysis and Method Validation

For the quantitative method development, five major representatives of coniferous wood lignans (secoisolariciresinol, matairesinol, pinoresinol, HMR, and nortrachelogenin) found in the majority of knotwood samples [6] were chosen as the target analytes. The analyses of the model solutions of various concentrations (up to 20 mg L^−1^) with the further construction of calibration plots (chromatographic peak area S vs. concentration C) showed good linearity (R^2^ > 0.999) in the range of at least two orders of magnitude (Table 2). Rather close response factors (calibration line slope *a*) differing by a maximum of two times were observed for all the analytes. The instrumental limits of detection (LODs) and quantification (LOQs) determined as the analyte concentrations providing signal-to-noise ratios of 3:1 and 10:1, respectively, were in the ranges of 0.08–0.29 (LOD) and 0.27–0.95 (LOQ) mg L^−1^. These values were additionally confirmed in the analysis of the sample with analyte concentrations close to the LOQ (Supplementary Figure S3). The attained sensitivity level is typical for LC-UV, and the obtained LOQs turned out to be noticeably lower than those reported in [11] for schisandrins (0.67 to 4.83 mg L^−1^).

As can be seen from Table 3 containing the results of intra- and inter-day assays, the achieved accuracy of the developed method was in the range of 91–109%, while the standard deviation (precision) did not exceed 13% at the LOQ level. The matrix effects were estimated by a spike recovery test using birch xylem extract as a matrix, which does not contain coniferous lignans. The obtained recovery values ranged from 82 to 98% for two levels of analyte concentrations (close to LOQ and 10 LOQ), indicating no substantial matrix interferences for all the analytes (Table 4). The effective elimination of the matrix effect was achieved by two-dimensional chromatographic separation of lignans from the matrix.

### 2.3. Analysis of Coniferous Knotwood Extracts

**Figure 2:**
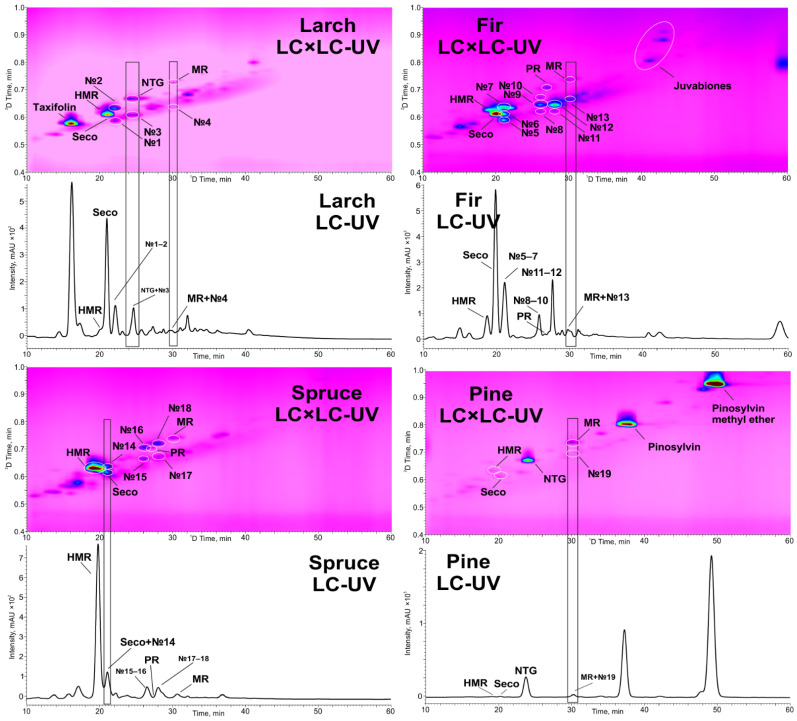
LC × LC-UV and LC-UV chromatograms (280 nm) of softwood knots extracts (MR—matairesinol, Seco—secoisolariciresinol, PR—pinoresinol, NTG—nortrachelogenin).

To test the developed approach, larch, fir, spruce, and pine knotwood acetone extracts were selected as the real samples. These objects are characterized by a complex chemical composition and contain a number of lignans. The obtained 2D chromatograms (Figure 2) demonstrate the presence of all the target analytes in a wide concentration range as well as other numerous components absorbing UV radiation (No. 1–19). The comparison of the one- and two-dimensional HPLC-UV chromatograms obtained on the same ^1^D column (Shim-pack XR-ODS II) clearly demonstrates the undoubted advantages of LC × LC.

The latter made it possible to separate the target analytes from other co-eluting in ^1^D compounds, for example, nortrachelogenin and unidentified compound No. 2, matairesinol and No. 4, matairesinol and No. 13, secoisolariciresinol and No. 14, and matairesinol and No 19. Moreover, the unidentified compounds, many of which belong to the lignan family, were also well separated (for example, No. 1–2; 5–7; 8–10; 11–12; 15–16; and 17–18). Despite the longer separation time (60 min), the LC × LC-UV approach has obvious advantages, even over LC-MS, both in terms of the cost of analysis and the elimination of matrix effects. The latter factor, together with similar absorption coefficients of various lignans in the UV region, can be the basis for the development of methods for the semi-quantitative standard-free determination of such compounds in plant extracts.

The results of the quantification of the target analytes (Table 5) are consistent with the literature data [6,30] and demonstrate the predominance of HMR (100 mg g^−1^), secoisolariciresinol (20 mg g^−1^), and nortrachelogenin (8 mg g^−1^) in spruce, fir and larch, and pine knotwood, respectively.

In addition to lignans, other phenolic compounds were also found in the studied samples and were tentatively identified on the basis of data in the literature [5,6], UV absorption spectra (Supplementary Figures S4–S6) [31,32,33], and retention times. These include taxifolin (^1^D 16.05 min, ^2^D 0.60 min) in larch, pinosylvin (^1^D 37.60 min, ^2^D 0.80 min), its methyl ether (^1^D 49.80 min, ^2^D 0.95 min) in pine, and three juvabiones (^1^D 41.05 min, ^2^D 0.81 min; ^1^D 43.20 min, ^2^D 0.88 min; ^1^D 43.20 min, ^2^D 0.91 min) in fir knotwood extracts.

## 3. Materials and Methods

### 3.1. Chemicals and Reagents

Commercially available standards of pinoresinol (≥95%), secoisolariciresinol (≥95%), and matairesinol (≥85%) were purchased from Sigma-Aldrich (St. Louis, MO, USA). 7-hydroxymatairesinol (≥98%) and nortrachelogenin (≥98%), which are poorly available as high-purity commercial preparations, were obtained in our laboratory from knotwood extracts by preparative liquid chromatography (Section 3.3).

Acetonitrile (HPLC gradient grade, Khimmed, Saint Petersburg, Russia), formic acid (≥96%, Sigma-Aldrich, St. Louis, MO, USA), and “type I” Milli-Q high-purity water were used for the preparation of the mobile phase. Methanol (high-purity grade, Khimmed, Saint Petersburg, Russia) was used for the preparation of the sample solutions. Hexane (chem. pure grade, Khimmed, Saint Petersburg, Russia) and acetone (pure, Vershina, Vsevolozhsk, Russia) were used for Soxhlet extraction.

The stock solutions of lignans in methanol (500 mg L^−1^) were prepared from accurately weighed samples and stored at 4 °C for no longer than one week. The model and calibration solutions of the analytes were prepared immediately before the analyses by mixing and successive dilutions of the stock solutions with methanol.

### 3.2. Plant Materials and Extraction

Four coniferous tree species were chosen as a source of plant material in our study—Norway spruce (*Picea abies*), Siberian fir (*Abies sibirica*), Scotch pine (*Pinus sylvestris*), and Larch (*Larix sibirica*). The tree trunks were harvested in boreal forests of the European North of Russia in the following locations: 64°76′ N 40°80′ E (pine), 64°28′ N 40°76′ E (spruce) 61°77′ N 42°47′ E (fir), and 61°16′ N 42°54′ E (larch). The parts with large knots were cut from at least five trunks of each species and immediately delivered to the laboratory. The knotwood samples were taken from the inner (inside the trunk) part of the knots by drilling and were carefully averaged. The obtained knotwood shavings were vacuum dried at 40 °C overnight and then milled in a ZM 200 centrifugal mill (Retsch, Haan, Germany) to a particle size of <1 mm.

Soxhlet extraction of the prepared sawdust was carried out according to a previously developed procedure [6]. The dried material (~10 g) was extracted using hexane for 8 h to remove lipids and resin. After air-drying, the plant material was subjected to exhaustive extraction using acetone for 8 h. The obtained acetone extracts were evaporated on a rotary evaporator RV 10 (IKA, Königswinter, Germany) to dryness. The attained yields of the extractive substances were larch—11.2%, fir—16.8%, spruce—16.3%, and pine—3.85%. The birch (*Betula pendula*) xylem extract was used for the estimation of the matrix effects (hardwood is supposed to be lignan-free).

### 3.3. Isolation and Characterization of Hydroxymatairesinol and Nortrachelogenin

Since 7-hydroxymatairesinol (HMR) and nortrachelogenin sharply predominate in spruce and pine knotwood extracts and can be effectively separated from matrix compounds [34], their preparations of sufficient purity were obtained by preparative HPLC. Chromatographic separations were carried out at a temperature of 40 °C using a semipreparative-scale Nucleodur C18 Gravity column (Macherey-Nagel, Duren, Germany), 250 × 21 mm, 5 μm particle size, on an LC-20 preparative HPLC system (Shimadzu, Kyoto, Japan) consisting of two LC-20AP pumps with high-pressure gradient formation, a vacuum degasser, a CTO-20A column thermostat, an SPD-M20A high-flow diode array UV-VIS detector, and an FRC-10A fraction collector. The system was controlled using LabSolutions software v. 5.54 (Shimadzu, Kyoto, Japan). The mobile phase was a mixture of components A (0.1% aqueous solution of formic acid) and B (acetonitrile with 0.1% of formic acid) with a total flow rate of 21.0 mL min^−1^. The following gradient elution program was applied: 0–20 min—25% B; 20–25 min—linear ramp to 100% B, held for 10 min. The total separation time was 35 min.

The 50 mg samples of the dried knotwood extracts were redissolved in 2 mL of 50% aqueous methanol, centrifuged, and manually injected (2 mL) into the HPLC system. The target fractions of HMR and nortrachelogenin were collected in the periods of 9.50–10.50 and 16.20–17.50 min, respectively (Supplementary Figure S7) and then evaporated on a rotary evaporator to dryness and redissolved in 1 mL of methanol. Their purity was estimated in an additional chromatographic analysis using the same analytical HPLC system as for the lignan analysis (Section 3.4). The content of the target compound (%) was calculated as a ratio of the corresponding chromatographic peak area and the total area of all the peaks on the chromatogram.

The structures of the isolated individual compounds were confirmed by their ^1^H and ^13^C NMR spectra registered at 25 °C in CD_3_OD on an AVANCE III NMR spectrometer (Bruker, Ettlingen, Germany), with an operational frequency of 600 MHz (^1^H). The following parameters were applied: (i) ^1^H NMR: zg30 sequence, acquisition time—1.4 s, relaxation delay—1 s, number of data points—64,000, number of scans—8, spectrum window width ~15 ppm; (ii) ^13^C NMR: zgig30 sequence, pulse width—12 ms; relaxation delay—2 s; number of data points—64,000, number of scans—1024, spectrum window width ~240 ppm. The solvent signal was used as an internal standard (dC/dH 49.15/3.31 ppm).

The characteristics of the obtained preparations were as follows. 7-Hydroxymatairesinol: light beige powder; purity: 98%; UV (MeOH): λ max 228, 280 nm; ^1^H NMR (600 MHz, CD_3_OD) and ^13^C NMR (150 MHz, CD_3_OD). The ^1^H and ^13^C NMR data are presented in Supplementary Tables S1 and S2, Figures S8 and S9.

Nortrachelogenin: light beige powder; purity: 98%; UV (MeOH): λ max 228, 280 nm; ^1^H NMR (600 MHz, CD_3_OD) and ^13^C NMR (150 MHz, CD_3_OD). The ^1^H and ^13^C NMR data are presented in Supplementary Tables S1 and S2, Figures S10 and S11.

### 3.4. Comprehensive Two-Dimensional Liquid Chromatography

**Figure 3:**
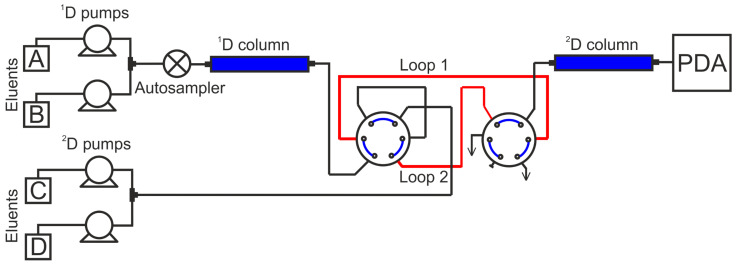
Schematic diagram of the LC × LC-UV system.

A comprehensive two-dimensional liquid chromatography system, Nexera-e (Shimadzu, Kyoto, Japan), was used for the one- and two-dimensional analytical separations and consisted of four LC-30AD pumps with gradient formation on the high-pressure side, two five-channel degasser units DGU-A5, an LC-30AC autosampler, a CTO-30A column thermostat, two high-speed/high-pressure six-port switching valves, equipped with two sampling loops (volume 50 μL) and an SPD-M20A diode array detector. A schematic diagram of the LC × LC system is presented in Figure 3. The controlling of the HPLC system as well as the data collection and processing were carried out using the LabSolutions 5.65 software package (Shimadzu, Kyoto, Japan).

Knotwood extracts (1.00 mg) were dissolved in 1 mL of methanol, centrifuged, and injected into the LC × LC system. Separation was performed on the corresponding ^1^D and ^2^D columns with flow rates of 0.05 and 2.0 mL min^−1^, respectively. The effluent from the first column was transferred to the loop of the flow-switching valve. Every 60 s (modulation time), the collected 50 μL portion of the ^1^D column effluent was injected into the ^2^D column by switching the valve. ChromSquare 2.2 (Chromaleont, Messina, Italy) was used for the system control and the construction of the LC × LC chromatograms. The chromatographic separations were carried out at 40 °C. The detection was performed at 280 nm. The injection volume was 4 μL.

The following chromatographic columns were used in the ^1^D separations: Shim-pack XR-ODS II, 3 × 50 mm, 2.2 μm particle size (Shimadzu, Kyoto, Japan); Nucleodur C18 Isis, 2.0 × 150 mm, 1.8 μm particle size (Macherey-Nagel, Duren, Germany); Nucleodur PFP, 2.0 × 150 mm, 1.8 μm particle size (Macherey-Nagel, Duren, Germany); Nucleodur PolarTec, 2.0 × 150 mm, 1.8 μm particle size (Macherey-Nagel, Duren, Germany). In the ^2^D separations, the following columns were used: Nucleodur PFP, 4.6 × 30 mm, 1.8 μm particle size (Macherey-Nagel, Duren, Germany) and Nucleodur C18 Pyramid, 4.6 × 50 mm, 1.8 μm particle size (Macherey-Nagel, Duren, Germany).

## 4. Conclusions

Comprehensive two-dimensional liquid chromatography on reversed stationary phases with different chemistries ensures the efficient separation of coniferous wood lignans in plant extracts in the gradient elution mode. The highest “orthogonality” and best separation are achieved on a combination of non-polar and polar hydroxy group embedded octadecyl stationary phases in the first and second dimensions, respectively. On this basis, a novel method for the quantification of the five lignans (secoisolariciresinol, matairesinol, pinoresinol, HMR, and nortrachelogenin) in knotwood extracts by LC × LC with UV diode array detection was developed and validated as an alternative to LC-MS assays. The attained LOQs of the target analytes ranged from 0.27 to 0.95 mg L^−1^, and the calibration plots were linear in the concentration range covering at least two orders of magnitude. Testing the developed method on coniferous (larch, fir, spruce, and pine) knotwood extracts demonstrated the high selectivity of the analysis, which allowed the separation and quantification of the target analytes and matrix components co-eluting in one-dimensional HPLC. The proposed approach can be used to develop and improve methods for the determination of lignans in various objects. Further studies should be focused on the hyphenation of LC × LC separation with mass spectrometric detection for non-target screening, identification, and the highly sensitive determination of lignans and their derivatives in plant extracts.

## Figures and Tables

**Table 1:** Orthogonality (A_O_) and regression coefficient (R^2^) parameters for different combinations of stationary phases in the LC × LC separation of lignan-rich larch knotwood extract.

Stationary Phase		A_O_ (%)	R^2^
^1^D	^2^D		
Shim-pack XR-ODS II	Nucleodur C18 Pyramid	39	0.84
Nucleodur C18 Isis	Nucleodur C18 Pyramid	34	0.92
Nucleodur PFP	Nucleodur C18 Pyramid	40	0.85
Nucleodur PolarTec	Nucleodur PFP	38	0.88
Shim-pack XR-ODS II	Nucleodur PFP	38	0.87
Nucleodur PolarTec	Nucleodur C18 Pyramid	36	0.88

**Table 2:** Calibration dependences (S = aC + b) for the area of chromatographic peak versus analyte concentration, LODs, LOQs, and retention times of analytes.

Analyte	Retention Time, min		Linear Range, mg L^−1^	a	b	R^2^	LOD, mg L^−1^	LOQ, mg L^−1^
	^1^D	^2^D						
HMR	18.6	0.62	0.45–20	15389	−4511	>0.999	0.13	0.44
Secoisolariciresinol	20.6	0.61	0.35–20	20407	−5854	>0.999	0.16	0.54
Nortrachelogenin	23.7	0.67	0.36–20	16557	−1944	>0.999	0.11	0.37
Pinoresinol	27.7	0.70	0.29–20	37764	−10824	>0.999	0.29	0.95
Matairesinol	30.7	0.73	0.31–20	21436	−2998	>0.999	0.08	0.27

**Table 3:** Method accuracy and precision estimated in intra-day and inter-day assays of the model solution of lignans.

Analyte	Concentration, mg L^−1^	Intra-Day Assay (n = 6)			Inter-Day Assay (n = 6)		
		Found, mg L^−1^	Accuracy, %	Precision, %	Found, mg L^−1^	Accuracy, %	Precision, %
HMR	0.50	0.46 ± 0.03	91	4.66	0.45 ± 0.02	90	3.14
Secoisolariciresinol	0.50	0.51 ± 0.08	102	11.1	0.46 ± 0.04	92	6.11
Nortrachelogenin	0.50	0.49 ± 0.05	97	7.29	0.45 ± 0.07	89	11.1
Pinoresinol	1.00	1.00 ± 0.02	99	1.43	0.51 ± 0.09	101	12.6
Matairesinol	0.50	0.54 ± 0.07	107	9.25	0.55 ± 0.07	109	9.08

**Table 4:** Matrix effect estimated by the spike recovery test.

Analyte	Spiked, mg L^−1^	Found, mg L^−1^	Recovery, %
HMR	1.0	0.82 ± 0.06	82
	10	8.4 ± 0.8	84
Secoisolariciresinol	1.0	0.82 ± 0.15	82
	10	9.0 ± 0.4	90
Nortrachelogenin	1.0	0.85 ± 0.08	85
	10	9.3 ± 0.7	93
Pinoresinol	1.0	8.8 ± 0.7	88
	10	9.8 ± 0.2	98
Matairesinol	1.0	8.9 ± 1.0	89
	10	9.0 ± 0.4	90

**Table 5:** The content of lignans (mg g^−1^, recalculated for the oven-dried plant material) in softwood knots (n = 2, *p* = 0.95).

Analyte	Larch	Fir	Spruce	Pine
HMR	1.2 ± 0.2	0.17 ± 0.04	100 ± 10	0.23 ± 0.03
Secoisolariciresinol	17 ± 6	20 ± 2	6.1 ± 0.7	0.24 ± 0.08
Nortrachelogenin	3.3 ± 0.4	-	-	8.0 ± 0.9
Pinoresinol	-	0.78 ± 0.14	0.31 ± 0.05	-
Matairesinol	0.55 ± 0.07	0.75 ± 0.17	1.0 ± 0.2	0.65 ± 0.13

## Data Availability

The data presented in this study are available in the article and Supplementary Materials.

## Supplementary Materials

The following supporting information can be downloaded at https://www.mdpi.com/article/10.3390/molecules28248114/s1, Figure S1: LC × LC-UV chromatograms (280 nm) of larch knotwood extract recorded on different 1D and 2D column combinations; Figure S2: Scaled UV spectra of lignans; Figure S3: LC × LC-UV chromatogram (280 nm) of analyte model mixture (1.0 mg L^−1^).; Figure S4: UV spectrum of the component of the larch knotwood extract with ^1^D Time 16.05 min and ^2^D Time 0.58 min; Figure S5: UV spectra of the components of pine knotwood extract; Figure S6: UV spectra of the components of fir knotwood extract; Figure S7: Preparative LC-UV chromatograms (280 nm) of extract; Figure S8: ^1^H NMR spectrum of hydroxymatairesinol (Methanol-d_4_); Figure S9: ^13^C NMR spectrum of hydroxymatairesinol (Methanol-d_4_); Figure S10: ^1^H NMR spectrum of nortrachelogenin (Methanol-d_4_); Figure S11: ^13^C NMR spectrum of nortrachelogenin (Methanol-d_4_). Table S1: ^1^H NMR (600 MHz) Spectroscopic Data of hydroxymatairesinol and nortrachelogenin; Table S2: ^13^C NMR (150 MHz) Spectroscopic Data of hydroxymatairesinol and nortrachelogenin.
