# Supplementary material for: Analysis of Softwood Lignans by Comprehensive Two-Dimensional Liquid Chromatography
Source: Molecules. 2023 Dec 15;28(24):8114. doi: 10.3390/molecules28248114 (PMC10745517; doi:10.3390/molecules28248114)
Supplement: Supplementary file 1 [file molecules-28-08114-s001.zip › molecules-2758365-supplementary.pdf]

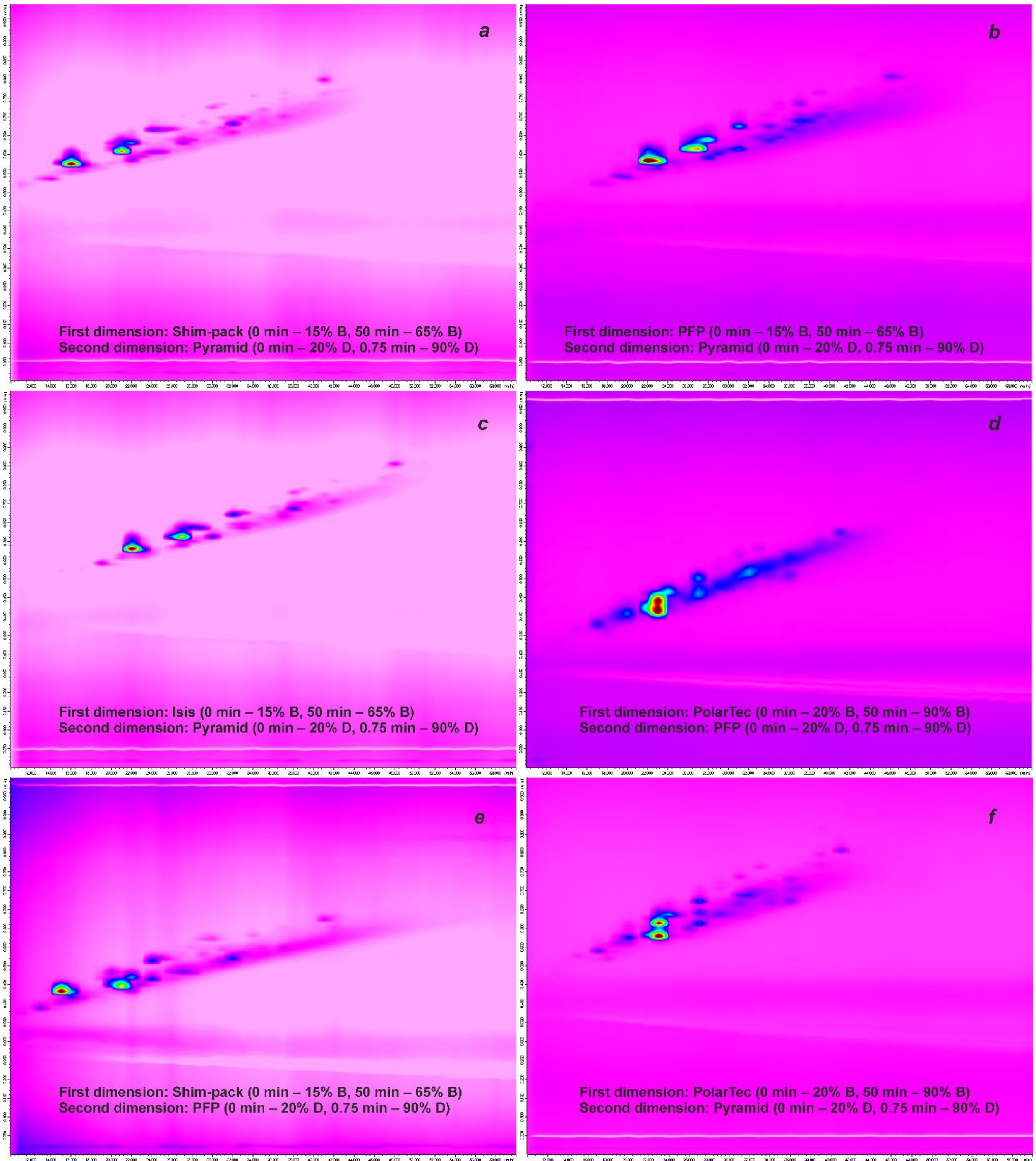

Figure S1. LC×LC-UV chromatograms (280 nm) of larch knotwood extract recorded on different ^{1}D and ^{2}D column combinations.

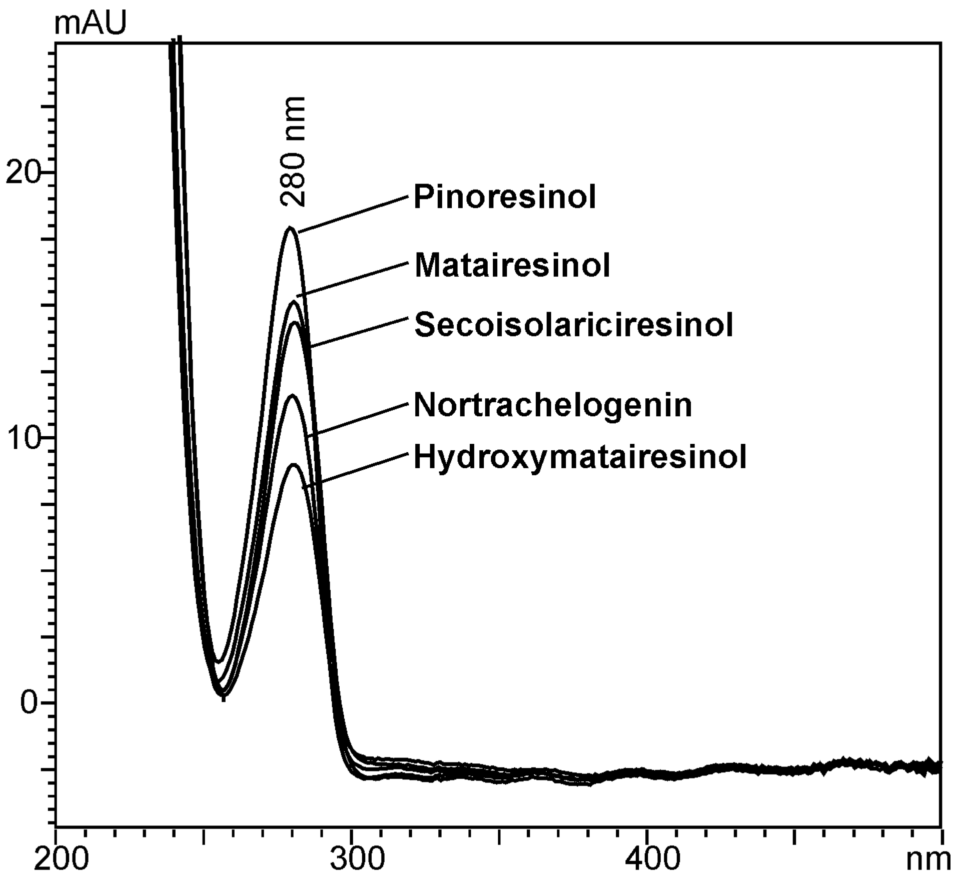

Figure S2. Scaled UV spectra of lignans.

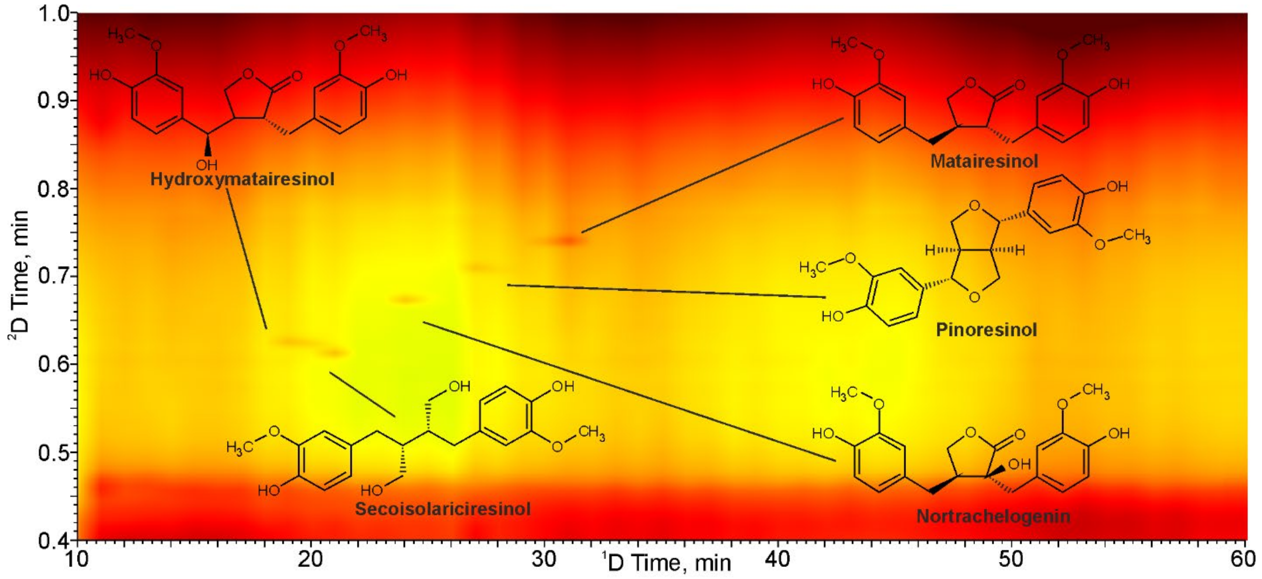

Figure S3. LC×LC-UV chromatogram (280 nm) of analytes model mixture (1.0 mg L^{-1}).

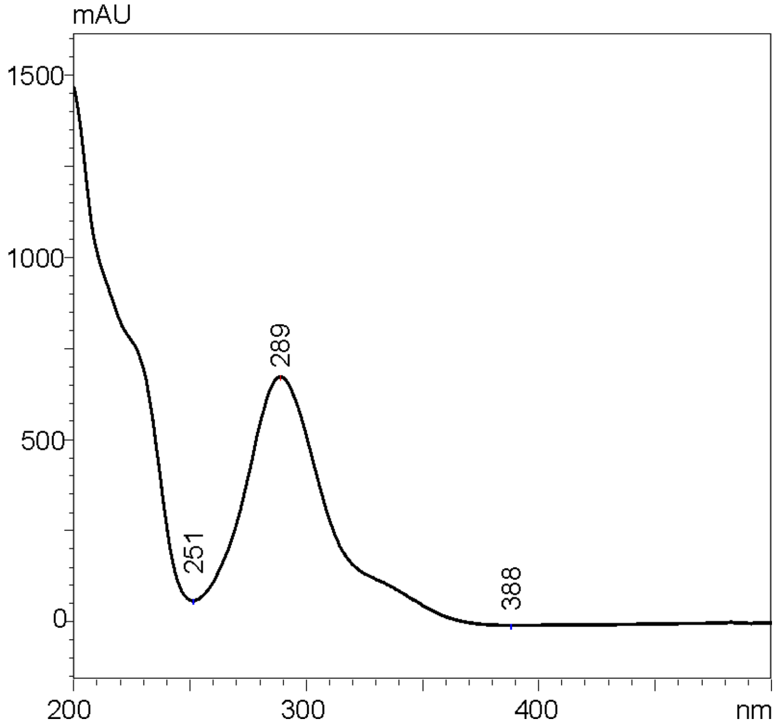

Figure S4. UV spectrum of the component of the larch knotwood extract with [1]D Time 16.05 min and [2]D Time 0.58 min.

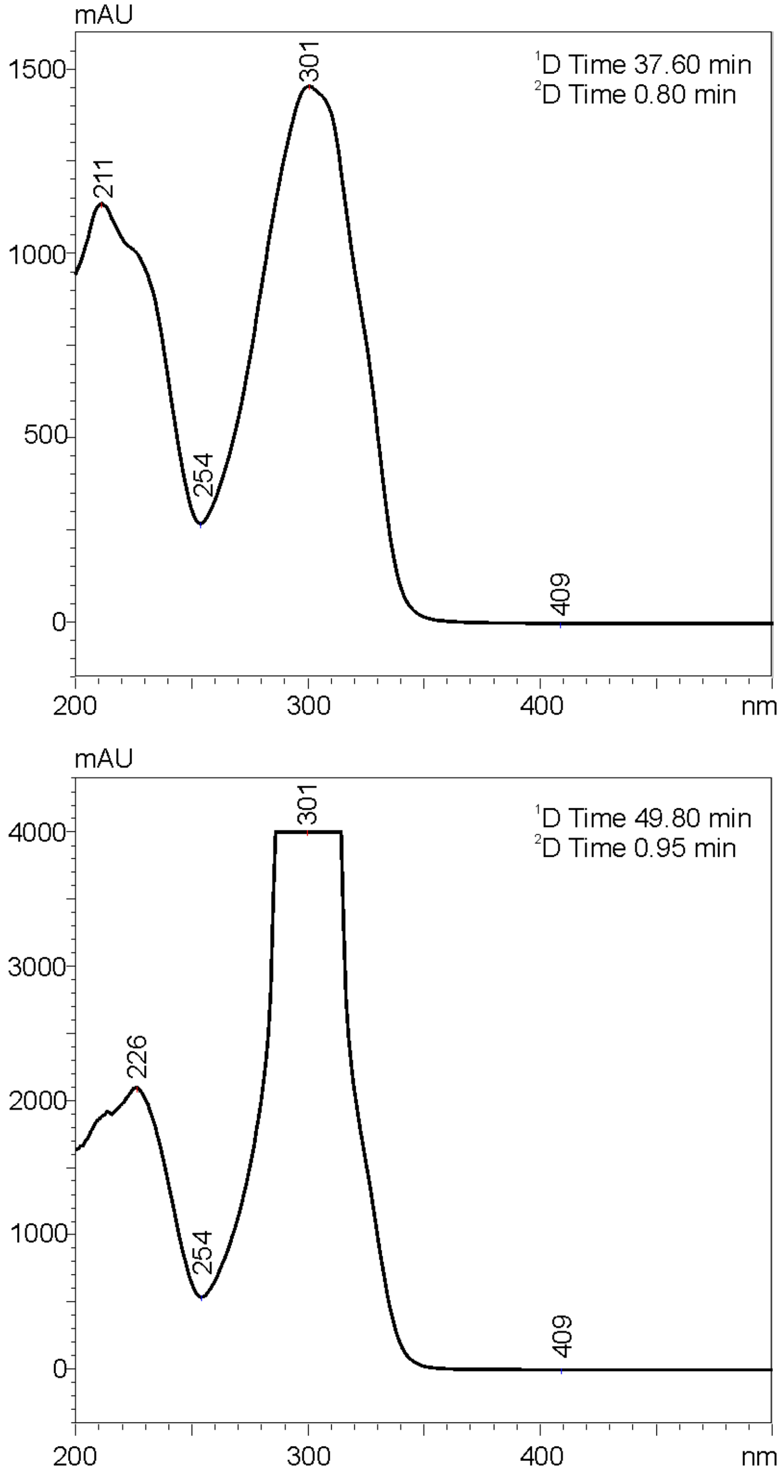

Figure S5. UV spectra of the components of pine knotwood extract.

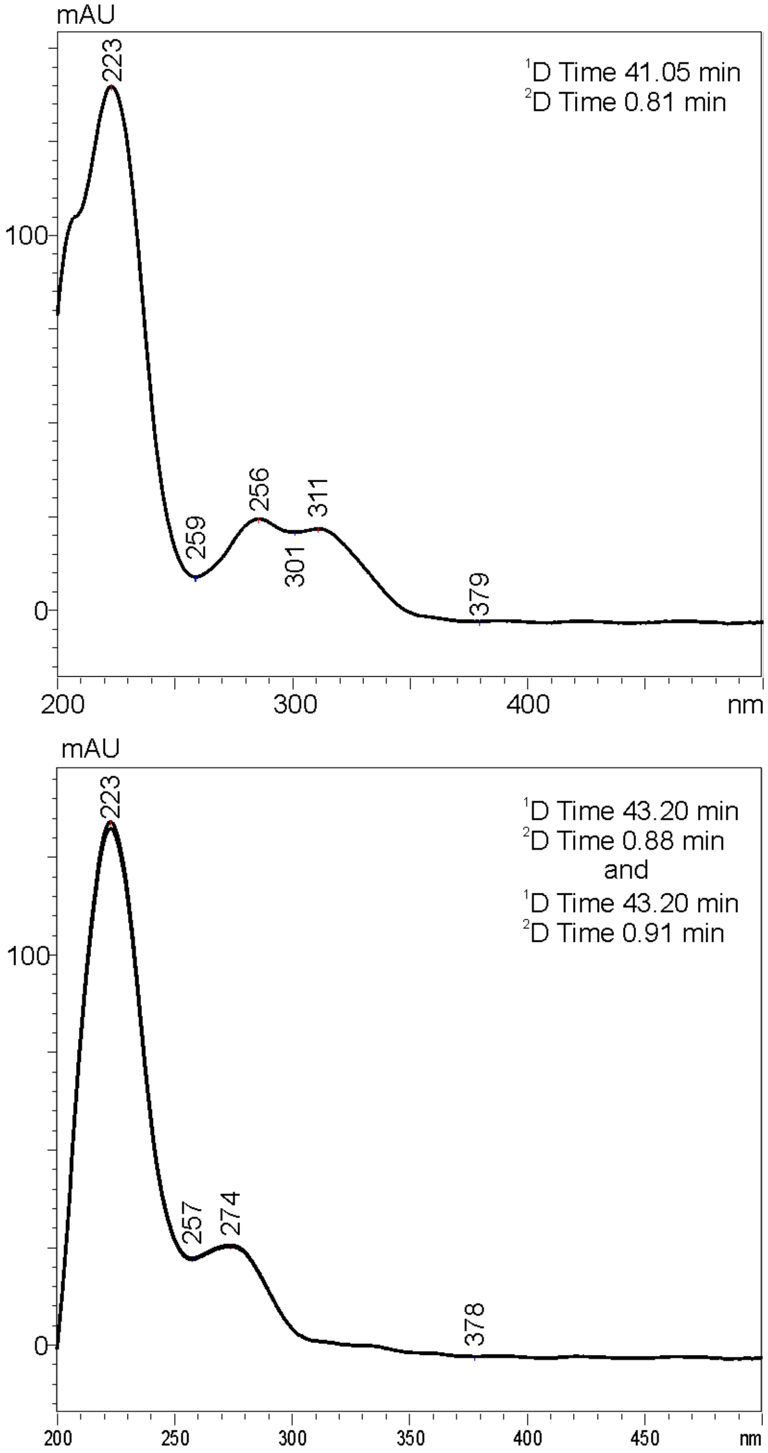

Figure S6. UV spectra of the components of fir knotwood extract

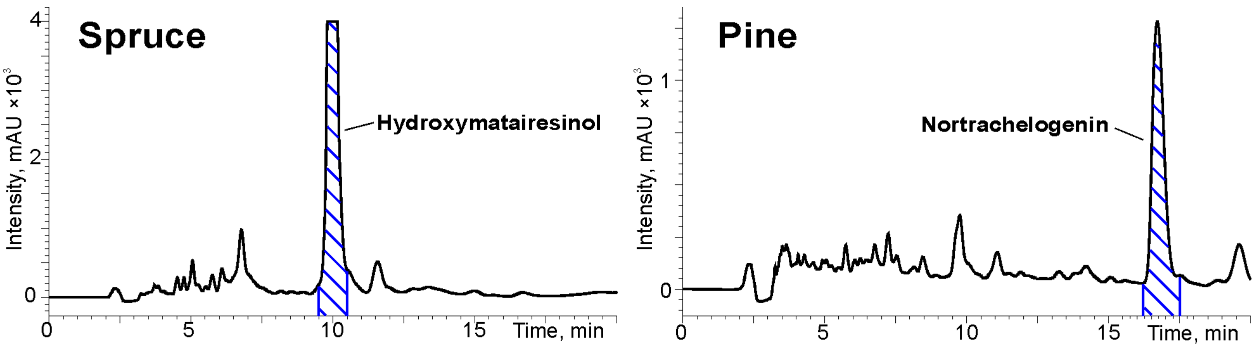

Figure S7. Preparative LC-UV chromatograms (280 nm) of extracts.

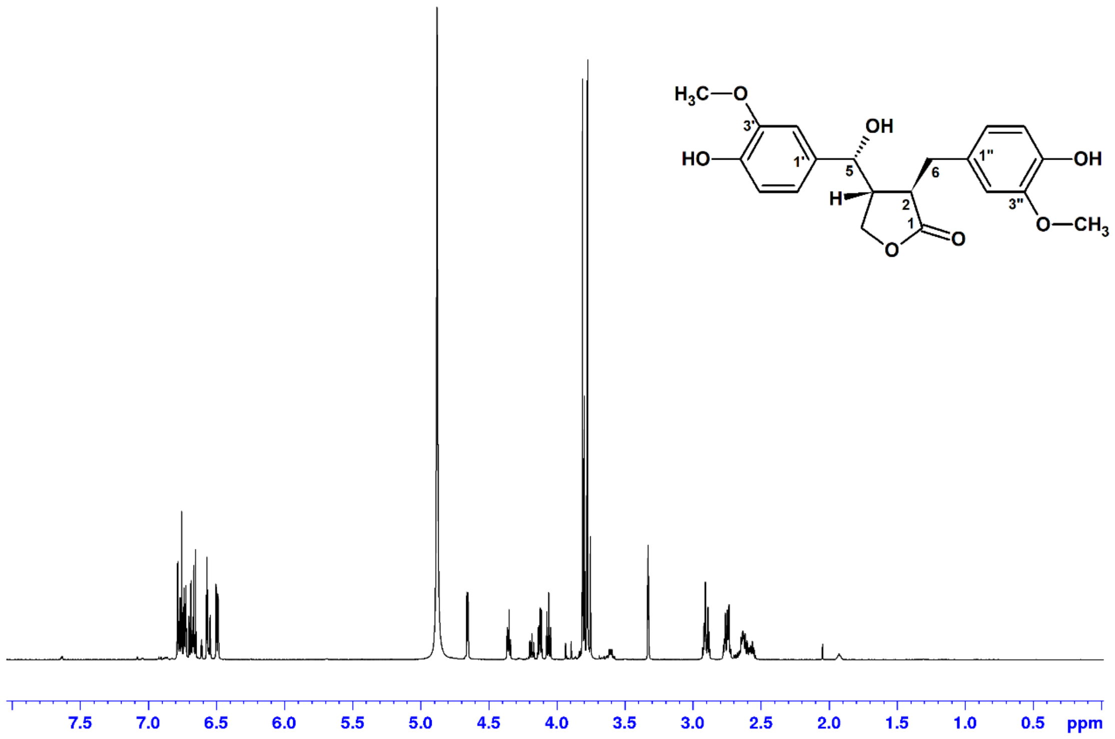

Figure S8. ^{1}H NMR spectrum of hydroxymatairesinol (Methanol-d_4).

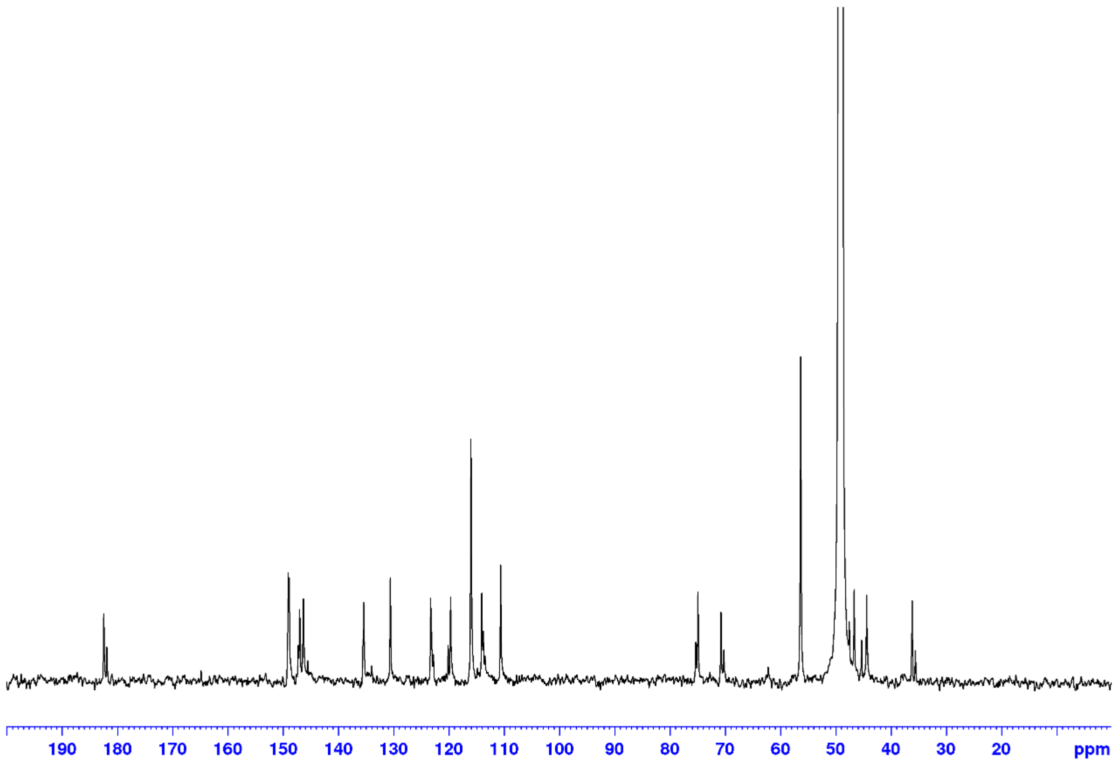

Figure S9. ^{13}C NMR spectrum of hydroxymatairesinol (Methanol-d_4).

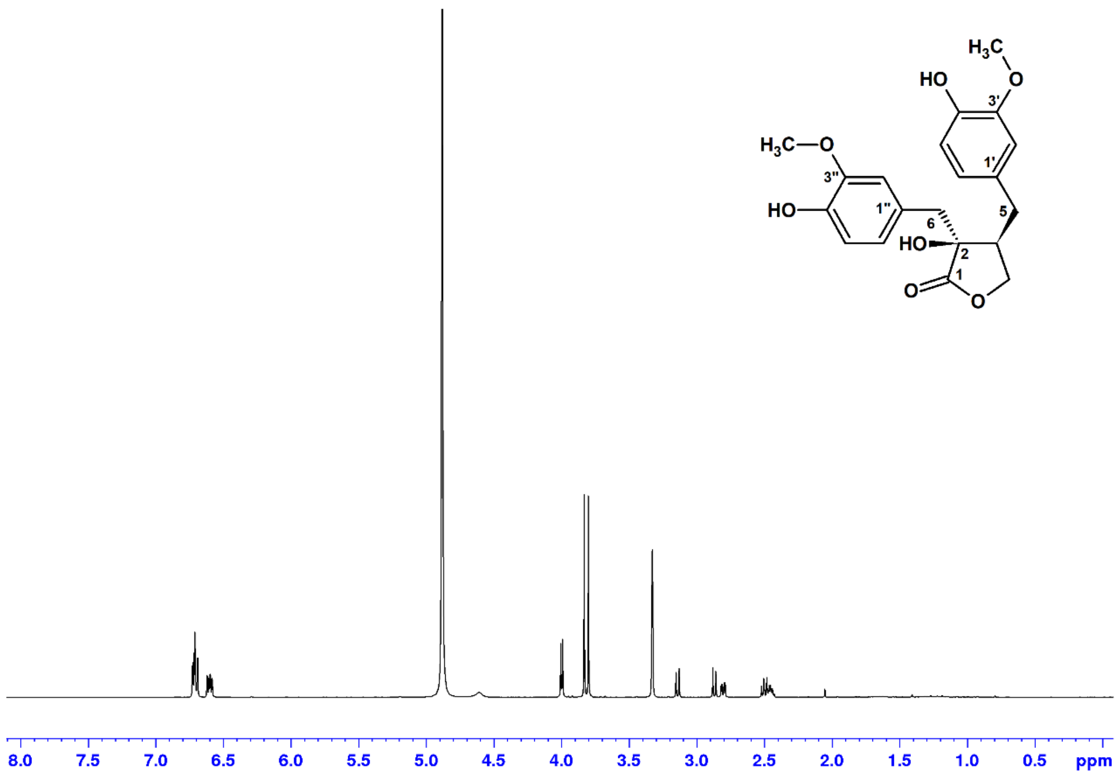

Figure S10. ^{1}H NMR spectrum of nortrachelogenin (Methanol-d_4).

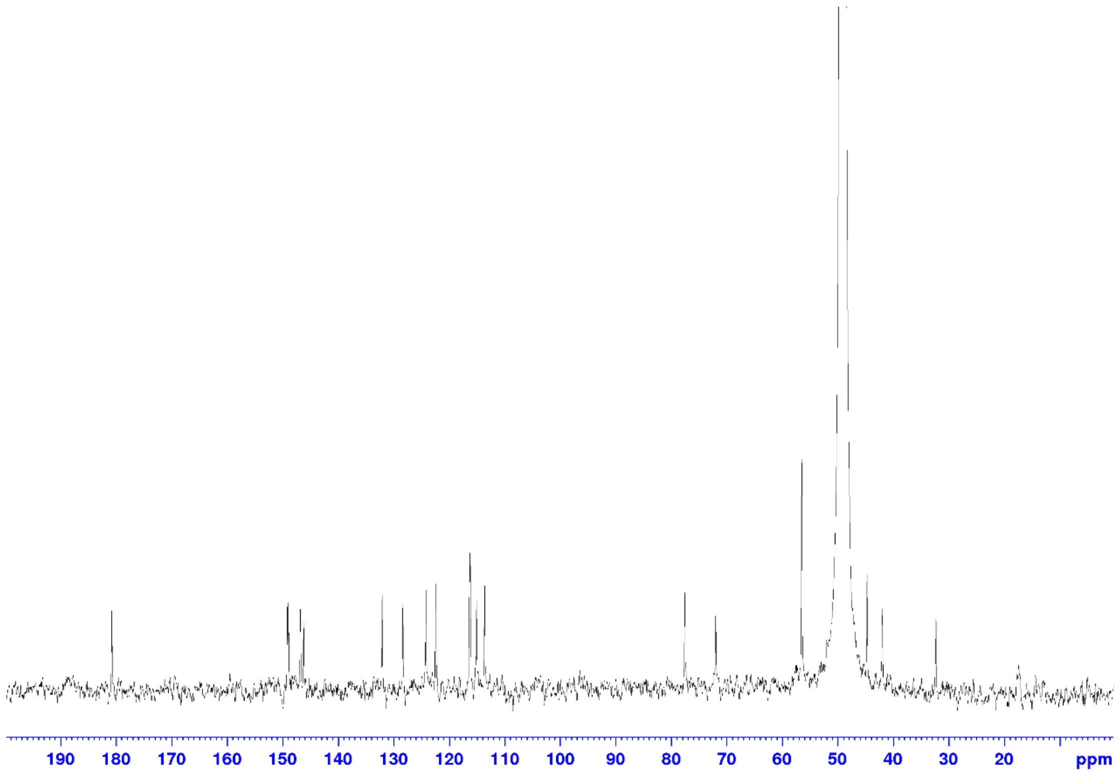

Figure S11. ^{13}C NMR spectrum of nortrachelogenin (Methanol-d_4).

Table S1. ^{1}H NMR (600 MHz) Spectroscopic Data of hydroxymatairesinol (1) and nortrachelogenin (2).

proton	1	1 (isomer)	2
1	-	-	-
2	2.89 m	2.72 m	-
3	2.62 m	2.55 m	2.44 m
4	4.10 t (8.75), 4.04 t (10.09)	4.24 m	3.98 d (8.25)
5	4.64 d (5.23)	4.34 d (7.06)	2.65 ddd (13.34; 4.81)
6	2.8	2.8	2.99 dd (13.48)
1'	-	-	-
2'	6.77 m	6.71 m	6.67 m
3'	-	-	-
4'	-	-	-
5'	6.74 m	6.67 m	6.7 m
6'	6.71 m	6.67 m	6.6 m
1''	-	-	-
2''	6.55 m	6.53 m	6.69 m
3''	-	-	-
4''	-	-	-
5''	6.64 m	6.64 m	6.7 m
6''	6.47 m	6.47 m	6.57 m
OCH_3-3'	3.79 s	3.79 s	3.78 s
OCH_3-3''	3.75 s	3.75 s	3.81 s

Table S2. ^{13}C NMR (150 MHz) Spectroscopic Data of hydroxymatairesinol (1) and nortrachelogenin (2).

carbon	1	1 (isomer)	2
1	182.04	181.62	180.53
2	44.06	45.05	77.34
3	46.35	47.27	44.58
4	70.33	69.76	71.55
5	74.61	75.05	32.05
6	35.89	35.89	41.8
1'	135.13	133.64	131.87
2'	110.39	110.25	113.42
3'	148.78	148.78	148.65
4'	146.68	147.15	146.08
5'	115.73	115.73	115.84
6'	119.44	119.84	122.24
1''	113.19	113.19	128.15
2''	113.62	113.59	114.83
3''	148.59	148.59	148.87
4''	146.02	146.02	146.59
5''	115.68	115.68	115.84
6''	123.03	123.03	123.97
OCH_3-3'	55.84	55.84	56.01
OCH_3-3''	55.84	55.84	55.93
